# A Canonical Scheme of Bottom-Up and Top-Down Information Flows in the Frontoparietal Network

**DOI:** 10.3389/fncir.2021.691314

**Published:** 2021-08-12

**Authors:** Eun Jung Hwang, Takashi R. Sato, Tatsuo K. Sato

**Affiliations:** ^1^Stanson Toshok Center for Brain Function and Repair, Brain Science Institute, Rosalind Franklin University of Medicine and Science, North Chicago, IL, United States; ^2^Cell Biology and Anatomy, Chicago Medical School, Rosalind Franklin University of Medicine and Science, North Chicago, IL, United States; ^3^Department of Neuroscience, Medical University of South Carolina, Charleston, SC, United States; ^4^Department of Physiology, Monash University, Clayton, VIC, Australia; ^5^Neuroscience Program, Biomedicine Discovery Institute, Monash University, Clayton, VIC, Australia; ^6^PRESTO, Japan Science and Technology Agency, Kawaguchi, Japan

**Keywords:** parietal cortex, frontal cortex, long-range connectivity, projection-specific, projection neurons, inter-area communication, goal-directed behavior, Alzheimer’s disease

## Abstract

Goal-directed behavior often involves temporal separation and flexible context-dependent association between sensory input and motor output. The control of goal-directed behavior is proposed to lie in the frontoparietal network, but the computational architecture of this network remains elusive. Based on recent rodent studies that measured and manipulated projection neurons in the frontoparietal network together with findings from earlier primate studies, we propose a canonical scheme of information flows in this network. The parietofrontal pathway transmits the spatial information of a sensory stimulus or internal motor bias to drive motor programs in the frontal areas. This pathway might consist of multiple parallel connections, each controlling distinct motor effectors. The frontoparietal pathway sends the spatial information of cognitively processed motor plans through multiple parallel connections. Each of these connections could support distinct spatial functions that use the motor target information, including attention allocation, multi-body part coordination, and forward estimation of movement state (i.e., forward models). The parallel pathways in the frontoparietal network enable dynamic interactions between regions that are tuned for specific goal-directed behaviors. This scheme offers a promising framework within which the computational architecture of the frontoparietal network and the underlying circuit mechanisms can be delineated in a systematic way, providing a holistic understanding of information processing in this network. Clarifying this network may also improve the diagnosis and treatment of behavioral deficits associated with dysfunctional frontoparietal connectivity in various neurological disorders including Alzheimer’s disease.

## Introduction

Our behavior flexibly changes depending on the desired goal. Approaching the same intersection, we may prepare to turn left or right depending on our current goal (e.g., going to work vs. the supermarket). Faced with the same dessert at the supermarket, we may choose to put it in our shopping cart or not depending on our desired goal (e.g., entertaining guests at a party or losing weight). Brain areas supporting such flexible goal-directed behavior include the posterior parietal cortex (PPC), frontal premotor cortex (PMC), and prefrontal cortex (PFC), collectively forming the frontoparietal network (Uddin et al., 2019). Numerous imaging and electrophysiological studies in humans and non-human primates have implicated this network in a variety of cognitive processes underlying goal-directed behavior, such as decision-making, memory, attention, motor planning, and sensorimotor control (Corbetta, 1998; Andersen and Cui, 2009; Ptak et al., 2017; Violante et al., 2017; Battaglia-Mayer and Caminiti, 2019). Despite extensive research on single-neuron activity and functional magnetic resonance imaging (fMRI) signals across the frontoparietal network, however, the computational architecture of this multi-region network remains elusive and basic questions are largely unanswered. For example, what are the unique roles of different brain areas in the frontoparietal network, what kinds of information are exchanged between areas, and how does this network perform many related yet distinct functions? Most frontoparietal areas show highly complex and heterogeneous neural activity patterns, reflecting the associative nature of their functional roles. Furthermore, complex activity patterns are extremely similar among different brain areas, likely due to their convergent interactions via reciprocal connections. These similar and complex activity patterns mask the unique contributions of each area, making it difficult to infer the computational architecture, including the flow of information processing among different brain areas in the network.

One way to unravel the flow of information processing in a network is to identify the information content transmitted from one area to another. The direct characterization of inter-areal information flows requires the examination of the activity of projection neurons. For instance, an early study examined the activity of neurons projecting from the PPC to the frontal eye field (FEF) in the frontal lobe and found strong and prevalent visual activity in those neurons (Ferraina et al., 2002), implicating the PPC in early visual processing of sensory stimuli. Despite this early success, pathway-specific investigations of projection neurons have not been widely performed in the field due to the laborious and inefficient technical procedures required to identify projection neurons. Circumventing these technical difficulties, simultaneous multi-area activity recording and sophisticated signal analysis tools have been employed to infer the nature of inter-areal communication. For instance, inter-areal spike-field coherence analysis between the PPC and the dorsal premotor cortex (PMd) suggested that information flow from the frontal to the parietal cortex is enhanced during free-choice decision-making (Pesaran et al., 2008). Temporal lag analysis of spiking activity between the PPC and PMd suggested that cognitive rule-based action goal selection arises first in the frontal region and then in the parietal area (Westendorff et al., 2010). However, the observed coherence or temporal lag difference could be mediated by some of many possible indirect pathways connecting any two higher brain areas. Thus, direct investigation of projection neurons is still necessary to ascertain the route of the analytically inferred information transmission. Furthermore, examining task-dependent or learning-related changes in projection neurons can reveal the mechanisms underlying dynamic connectivity in this network (Pesaran et al., 2008; Suriya-Arunroj and Gail, 2019; Taghizadeh et al., 2020).

Recent scientific advances have made the study of projection neurons in the frontoparietal network more tractable. At the technical front, a diverse set of tools has been developed and become readily available for large ensemble recording and manipulation of projection neurons in specific pathways in rodents. At the biological front, studies have demonstrated that the frontoparietal areas in rodents, similar to primates, are involved in the control of goal-directed behavior (see later sections) (Sul et al., 2011; Harvey et al., 2012; Raposo et al., 2014; Hanks et al., 2015; Hwang et al., 2017). The similar functional properties of frontoparietal areas, together with advanced experimental tools, has created the unprecedented opportunity to directly interrogate information flows in the frontoparietal network in great detail. This Mini Review briefly describes the currently available pathway-specific investigation tools, highlights recent discoveries in the rodent frontoparietal network, proposes a canonical scheme of information flows in this network, and discusses the significance of investigating the frontoparietal network in Alzheimer’s disease (AD).

## Advancements in Studying Cortical Computations by Long-Range Connectivity

All cortical functions rely on long-range connectivity to exchange information between brain areas and support hierarchical and distributed neural processing (Felleman and Van Essen, 1991). Thus, to gain a holistic understanding of information processing in the brain network, it is crucial to identify the specific information that is transmitted by each connection or pathway. Such identification was traditionally achieved by identifying long-range projection neurons by eliciting antidromic spikes; electrical stimulation of axonal fibers at the projected area can cause antidromic spikes that can be confirmed by a collision test (Bishop et al., 1962). This antidromic identification approach was instrumental to the discovery of the information conveyed to the FEF both from the superior colliculus via the thalamus and from the parietal cortex (Ferraina et al., 2002; Sommer and Wurtz, 2002). However, this approach is technically arduous. Recent methodological advances have led to powerful tools for recording and manipulating the signals conveyed by long-range projection neurons, enabling us to investigate pathway-specific information flows more efficiently and precisely.

**FIGURE 1:**
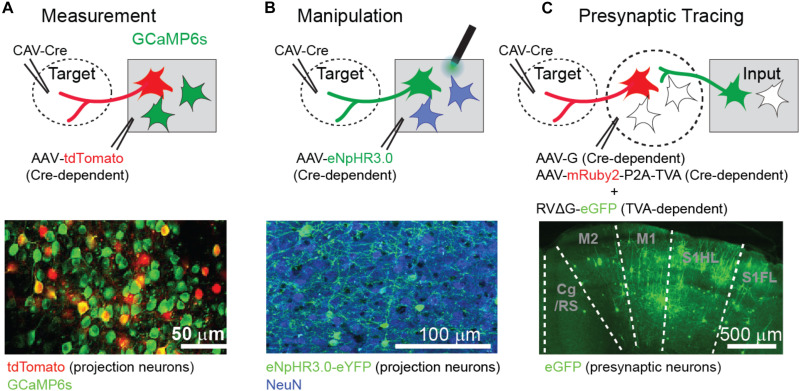
Approachese to investigate long-range projection neurons. **(A)** Identification of projection neurons by retrograde labeling and two-photon calcium imaging. For instance, projection neurons can be labeled with tdTomato by combining Cre recombinase expressing retrograde virus (CAV-Cre) and Cre-dependent tdTomato expressing adeno-associated virus (AAV). Bottom image shows tdTomato expression in projection neurons (red) together with GCaMP6s expression in cells driven by the CaMKII promotor (green). **(B)** Suppression of projection neurons by an inhibitory light-sensitive ion pump, eNpHR. Similar to **(A)**, projection neurons can express eNpHR by the combined viral approach. Bottom image shows eNpHR expression in projection neurons (green) and neuronal nuclear protein NeuN signal (blue) in all neurons. **(C)** Monosynaptic retrograde tracing of neurons presynaptic to projection neurons. The first step is to express Cre recombinase only in long-range projection neurons. The Cre expression can be anatomically confined by injecting retrograde virus into the target area. In the second step, the projection neurons must attain the capability for infection of modified rabies virus and its trans-synaptic spread by expressing a specific receptor (TVA) and rabies glycoprotein (G). These two proteins are expressed by Cre-dependent AAV in the Cre-expressing projection neurons (red neurons in **C**). The third step is to infect the projection neurons with pseudotyped, glycoprotein-deleted rabies virus, and to spread the rabies virus monosynaptically to presynaptic neurons brain-wide. Bottom image shows the remote input regions with GFP expression in long-range presynaptic neurons (green). All figures are modified from Hwang et al. (2019).

Currently, there are several ways to identify and record the activity of projection neurons. The first approach is to infect projection neurons with retrograde virus expressing light-sensitive ion channels (e.g., channelrhodopsin) and identify the spike activity upon direct activation of the cell bodies/somas (Lima et al., 2009) or antidromic activation of the projected axon terminals in a remote area (Sato et al., 2014; Economo et al., 2018). The second approach is to visually identify projection neurons by combining standard *in vivo* two-photon calcium imaging (Stosiek et al., 2003) with retrograde fluorescence labeling (Sato and Svoboda, 2010; Jarosiewicz et al., 2012; Chen et al., 2013; Yamashita et al., 2013; Hwang et al., 2019; Condylis et al., 2020; Figure 1A). The third approach is to monitor the activity of projection neurons by imaging their axon terminals (Petreanu et al., 2012; Glickfeld et al., 2013; Kwon et al., 2016; Itokazu et al., 2018). When projection neurons fire, spikes arrive at their axon terminals, evoking an increase in calcium concentration that can be detected by axonal calcium imaging.

Information flow from one area to another also can be manipulated in order to demonstrate its causal role in specific neural processing or even a behavior. Activation can be applied to test gain-of-function, and suppression can test loss-of-function. A pathway of interest can be activated optogenetically by illuminating axon terminals of projection neurons that are infected with virus expressing channelrhodopsin (Gradinaru et al., 2007). Alternatively, if the expression of channelrhodopsin is limited to the projection neurons by retrograde virus expression, light can be illuminated onto the somas of projection neurons (Znamenskiy and Zador, 2013). Similarly, specific pathways can be suppressed by expressing inhibitory light-sensitive pumps (Znamenskiy and Zador, 2013; Hwang et al., 2019). Illumination can be applied to somas or axons (Figure 1B). However, in some cases axonal optogenetic suppression causes anomalous synaptic activation, such as when using the light-activated proton pump eArch or ion channel GtACR (Mahn et al., 2016), requiring independent confirmation of results with somatic suppression. Axonal suppression can also be achieved using local chemogenetic approaches (Stachniak et al., 2014); however, this method is not ideal if the temporal specificity of the suppression is critical. A new promising approach for axonal suppression is to inhibit synaptic vesicle release via an inhibitory G protein-coupled receptor (Copits et al., 2021; Mahn et al., 2021).

Information flow from one area to another is carried by projection neurons, and the projection neurons, in turn, receive inputs from presynaptic neurons in multiple brain areas. Such a series of information flows can be examined by a new technical approach that can visualize presynaptic neurons of specific projection neurons (i.e., the “tracing the relationship between input and output” or TRIO method) (Schwarz et al., 2015). This method utilizes the pseudotyped, glycoprotein-deleted rabies virus (Wickersham et al., 2007; Figure 1C), which infects certain cells (starter cells) that have EnvA receptors (TVA), and then spreads to the presynaptic neurons of the starter cells only when the starter cells express glycoprotein separately. Both the starter cells and the glycoprotein expression can be limited to projection neurons by a combination of Cre-dependent expression of the helper components (TVA and glycoprotein) and retrograde expression of Cre recombinase in the projection neurons. As an example, the TRIO method has been utilized to demonstrate that neurons that project from the parietal cortex to the frontal secondary motor cortex (M2) receive enriched sensorimotor inputs, including those from the primary motor cortex and the primary somatosensory upper-body regions (coronal section in Figure 1C; Hwang et al., 2019). Although toxicity issues need to be further resolved, this strategy could be extended to measure or manipulate the activity of neurons presynaptic to the projection neurons by using rabies virus that expresses either activity sensors or light-sensitive ion channels (Reardon et al., 2016).

Some of these powerful techniques have been applied in pathway-specific investigations of the frontoparietal network, providing important clues into the computational architecture of this network, as detailed in the following sections.

## Parietofrontal Connectivity

What information is transmitted from the parietal to the frontal areas? Insights into this question may be gained by examining the known features of the primate and rodent PPC. The primate PPC is parceled into multiple subregions, each projecting to selective frontal premotor regions and organizing actions involving specific motor effectors; the parietal reach region (PRR) projects to the PMd for arm reaches, the lateral intraparietal area (LIP) projects to the FEF for oculomotor behavior, and the anterior intraparietal area projects to the ventral premotor cortex for hand grasping (Cavada and Goldman-Rakic, 1989; Andersen et al., 1990; Stanton et al., 1995; Johnson et al., 1996; Tanné-Gariépy et al., 2002). The PRR neurons are active during the delay period in a memory-guided reach task when the upcoming reaches are toward their preferred locations, and inactivation of these neurons deteriorates the accuracy of the reach endpoints (Snyder et al., 1997; Hwang et al., 2012). The PRR neurons exhibit sustained activity during the delay period, indicative of working memory (Snyder et al., 1997). Analogous, the LIP neurons show sustained activity during the planning period of an eye saccade and are spatially tuned for particular saccade directions (Gnadt and Andersen, 1988). Intriguingly, the LIP neurons are also activated by salient, task-irrelevant visual stimuli (i.e., distractors) and the salient pop-out target location in a visual target search task before the frontal cortex, suggesting that they play a role in bottom-up spatial attention as well (Bisley and Goldberg, 2003; Buschman and Miller, 2007; Ibos et al., 2013). Additionally, the LIP neurons track the strength of the sensory evidence in a perceptual decision-making task, suggesting that they reflect the accumulation of evidence that supports the choice of a saccade toward a preferred direction (Shadlen and Newsome, 2001). However, inactivation of the LIP has no effect on the perceptual decision-making that requires sensory evidence accumulation; thus, the causal role of the neural activity reflecting sensory evidence accumulation remains to be determined (Katz et al., 2016). The LIP neurons also reflect the probability or size of a reward associated with the planned saccade and the bias for the saccade associated with a higher reward (Platt and Glimcher, 1999; Glimcher, 2003; Dorris and Glimcher, 2004). Such action bias coding appears to be updated by tracking previous trial choice and outcome history (Seo et al., 2009), and might account for the finding that inactivation of the LIP increases an ipsilateral choice bias in a free choice task (Wilke et al., 2012; Christopoulos et al., 2015; Katz et al., 2016). As such, extensive primate research has established that the primate PPC is central for various aspects of goal-directed behavior including action-planning, working memory, attention, decision-making, and internally generated choice bias.

Compared to the primate PPC, the functional and anatomical properties of the rodent PPC are less well understood. Nevertheless, a number of recent studies revealed common key functions and connectivity in the PPC between the two species. First, similar to the primate PPC, the rodent PPC spans between the visual and somatosensory regions on the dorsal part of the cortex and project to the frontal areas such as the M2, orbitofrontal cortex, and anterior cingulate cortex (Hovde et al., 2018; Lyamzin and Benucci, 2019). The region comprising the rodent PPC mainly includes the areas referred to as V_A_, and part of V_RL_ and V_AM_ (Hovde et al., 2018; Lyamzin and Benucci, 2019). Second, the mouse PPC neuronal activity encodes upcoming choice in a memory-guided go/no-go task during the delay period, and the PPC activity showed intermediate properties between the visual cortex and frontal motor cortex in terms of the temporal profile and the extent of mixing sensory and motor response information (Goard et al., 2016). These activity patterns suggest a role of the rodent PPC in sensorimotor transformation and action planning. Third, the PPC single-neuron activity in mice during memory-guided navigation in a virtual T-maze encodes the upcoming turning choice, and inactivation of the PPC impairs memory-guided task performance (Harvey et al., 2012; Driscoll et al., 2017), suggesting that the rodent PPC is involved in working memory in a decision-making task. Fourth, in rats, PPC activity indicates the accumulation of sensory evidence for perceptual decision-making, but no behavioral effect is observed when this activity is inactivated, suggesting that evidence accumulation takes place outside of the PPC (Erlich et al., 2015; Hanks et al., 2015; Licata et al., 2017; Scott et al., 2017). This absence of a causal effect recapitulates the findings in primates. Finally, in both rats and mice, PPC activity encodes the previous trial choice, outcome, and stimulus information history that biases future choices, and perturbing that activity alters the history-dependent bias (Morcos and Harvey, 2016; Hwang et al., 2017; Akrami et al., 2018). Although some mechanistic details diverge such as the lack of sustained activity during a delay period in the rodent PPC, all of these functional properties of the rodent PPC resemble those of the primate PPC in similar task settings.

**FIGURE 2:**
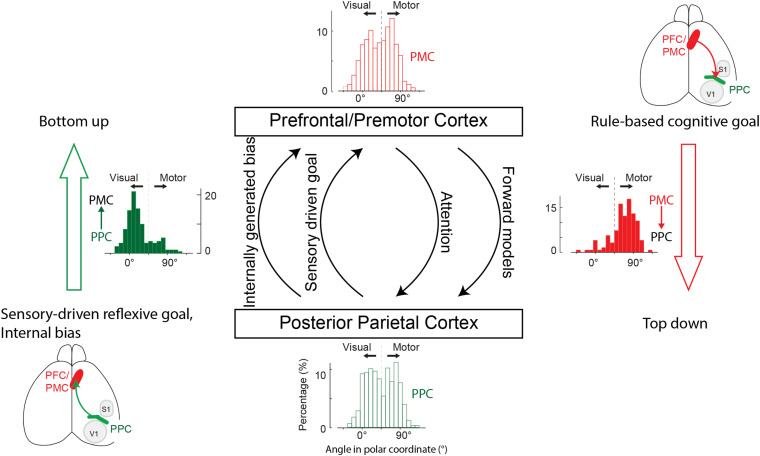
Proposed parallel information flows between the PFC/PMC and the PPC. The PPC sends PFC/PMC sensory-driven or internally biased target information for planning eye, limb, and whole-body movements. The PFC/PMC sends the PPC motor target information required for processing attention, forward models, and multibody coordination in parallel. Histograms represent the degree of sensory- vs. motor-target coding across all neurons in the PPC (bottom), axons projecting from the PPC to the PMC (left), all neurons in the PMC (top), and axons projecting from the PMC to the PPC (right). Coding preference for individual neurons or axons was represented as a polar angle in a two-dimensional plane in which the *x-* and *y*-axes correspond to visual and motor activity in a visually guided eye movement task in mice (Itokazu et al., 2018). Zero degree means purely visual activity and 90 degrees means solely motor activity.

Given these general functional analogies between species, the long-standing question of what information the PPC sends to the frontal areas may be efficiently examined in a rodent model using the aforementioned advanced techniques. Two recent studies in mice tackled this issue by measuring and manipulating the activity of neurons projecting from the PPC to the frontal area M2 in a mouse model. The first study examined the information flow from the PPC to the M2 by imaging the activity of PPC neuronal axons in the M2 during a visually guided eye movement task (Itokazu et al., 2018). The PPC neurons in this study reside in the areas traditionally referred to as the secondary visual cortex (V_RL_, V_A_, and V_AL_), which largely overlap with the posterior-lateral PPC where M2-projecting neurons are prevalent (Hwang et al., 2019; Lyamzin and Benucci, 2019). Itokazu et al. (2018) found that the general population of PPC neurons encodes a mixture of both the visual stimulus and movement target location information in a heterogeneous manner (bottom histogram in Figure 2), but M2-projecting neurons predominantly encode the location of the visual stimulus (left histogram in Figure 2). That is, the PPC sends the spatial information of the sensory stimulus to the M2. This result in mice is consistent with previous findings in macaques: (1) the visual response during a saccade task is more prevalent in PPC neurons projecting to the FEF than PPC neurons projecting to the superior colliculus (Ferraina et al., 2002), (2) neurons are more prevalent in the PPC than the PMd that preferentially represent a target for the stimulus-driven (i.e., visually guided), reflexive movement that coincides with the stimulus location (Westendorff et al., 2010), and (3) stimulus-driven spatial attention leading to an eye movement arises in the PPC before the FEF (Buschman and Miller, 2007; Ibos et al., 2013). Overall, the predominant transmission of the stimulus location information by the parietofrontal pathway fits well with the view that the PPC might feed into the frontal areas for the spatial information of a target for stimulus-driven reflexive and fixed movements (Andersen and Buneo, 2002).

What is the functional purpose of the information flow in the parietofrontal pathway? Although Itokazu et al. did not investigate the behavioral impact of inactivating PPC neurons projecting to the M2, a separate study examined these behavioral effects (Hwang et al., 2019). This study utilized retrograde virus to express inhibitory opsins selectively in projection neurons (Figure 1B). Hwang et al. (2019) found that inactivation of PPC neurons projecting to the M2 slows down upcoming movements and perturbs the movement trajectory in a forelimb-based visuomotor association task. This result is consistent with the known pathology that PPC inactivation or lesion causes optic ataxia, i.e., impaired spatial control of limb movements (Hwang et al., 2012; Andersen et al., 2014). Furthermore, Hwang et al. (2019) showed that inactivating a different path (the PPC to the striatum) did not impact the movement trajectory, underscoring that the PPC contributes to motor control specifically through its parietofrontal pathway. Thus, information transmitted by the parietofrontal pathway might be used to drive or shape motor programs in the frontal areas. It is noteworthy, however, that other rodent studies that inactivated the PPC did not find or report effects on movements (Harvey et al., 2012; Guo et al., 2014; Erlich et al., 2015; Goard et al., 2016). This discrepancy might be related to the fact that other studies examined movements such as licking, locomotion, and whole-body turning that may not require precise control over kinematics, while Hwang et al. examined finer forelimb movement trajectories. In addition, the inactivation effect in Hwang et al. (2019) was significant, yet subtle, such that only detailed kinematic analysis could reveal the induced changes on movements.

Hwang et al. (2019) also recorded the activity by combining retrograde labeling with two-photon calcium imaging (Figure 1A). This study found that PPC neurons projecting to the M2 encode the upcoming motor target location in the absence of a sensory stimulus, albeit significantly less than striatum-projecting PPC neurons, another type of major projection neuron. Using a computational model, Hwang et al. (2017) showed that mice in their task are biased toward one of the two motor targets before stimulus presentation, and representation of such internal biases underlies the coding of upcoming motor target location in PPC neurons (Hwang et al., 2017).

Taken together, these two mouse studies found that the parietofrontal pathway transmits the spatial information of both sensory stimuli and internal bias. These findings raise an intriguing question of whether the same population of PPC neurons encodes both the stimulus and internal bias information. Multiple scenarios can be conjectured. First, the two lines of input information, the external stimulus and internal bias, might be integrated in the same population of PPC neurons and utilize an overlapping pathway for motor control. Second, distinct PPC neurons might encode each type of information but converge and feed into the same M2 neurons. Third, distinct PPC neurons encoding each type of information might project to distinct subpopulations or subregions in the M2. This scenario suggests that the multiple parallel parietofrontal pathways might be segregated based on the type of information (i.e., internally versus externally driven information, Figure 2). In line with this scenario, the frontoparietal network in humans contains at least three parallel pathways, two of which are selectively activated by internal or external triggers (Uddin et al., 2019).

In addition to these scenarios, parallel pathways might be differentiated by the type of motor effector (i.e., forelimb vs. eye), similar to primates and humans, each controlling a specific effector such as saccade, reach, or grasp (Wise et al., 1997; Andersen et al., 2014; Battaglia-Mayer and Caminiti, 2019). Such parallel pathways likely exist given the necessity for somatotopic organization in the frontal motor areas. Consistent with this idea, an anatomical examination of the frontoparietal network in rats and mice found that all PPC subdivisions are strongly connected with the frontal area M2 in a topographically organized manner and largely reciprocate the densest input stems from the same areas (Sreenivasan et al., 2016; Zhang et al., 2016; Itokazu et al., 2018; Olsen et al., 2019). This anatomical configuration may exist to support the parallel organization of effector-specific frontoparietal connections. Clarifying those possibilities listed above will reveal the computational architecture of the network at a finer granularity.

## Frontoparietal Connectivity

What information is transmitted from the frontal to the parietal areas? Before delving into this question, we first review our current understanding of the frontal cortical regions that are strongly interconnected with the parietal regions in primates and rodents. In primates, the PMd is reciprocally connected with the PRR and the two areas show very similar neuronal response properties (Johnson et al., 1996; Wise et al., 1997; Musallam et al., 2004; Westendorff et al., 2010). In a delayed or memory-guided reach task, the PMd neurons show sustained activity before reaches to their preferred directions during the delay period, suggestive of action planning and working memory (Weinrich and Wise, 1982; Mitz et al., 1991; Crammond and Kalaska, 1994; Cisek et al., 2003; Churchland et al., 2006b; Gail et al., 2009). The delay-period activity of PMd neurons also encodes the speed and amplitude of upcoming movements, and inactivation of the PMd slows down the initiation of movements, reflecting its involvement in the control of motor programs (Churchland et al., 2006a; Churchland and Shenoy, 2007). The PMd neurons also encode the movement plan inferred from conditional sensorimotor associations (e.g., red and left vs. blue and right) (Mitz et al., 1991; Wise and Murray, 2000). PMd lesions impair such conditional sensorimotor associations (Passingham, 1988; Kurata and Hoffman, 1994), implicating the PMd in abstract rule-based action selection.

In parallel to the close link between the PMd and PRR for reaching movements, the primate FEF in the frontal lobe and the LIP are reciprocally connected for eye movements and attention (Bruce and Goldberg, 1985; Bruce et al., 1985; Stanton et al., 1989). Note that although the FEF is considered to be part of the PFC, it shows cytoarchitectural and functional properties reminiscent of the frontal motor areas. Unlike other PFC areas, the FEF has a relatively subtle layer 4, a dense population of large pyramidal neurons in layer 5, and a population of large layer 3 pyramidal neurons, similar to the primary motor cortical areas (Stanton et al., 1989). In addition, small electrical currents in the FEF robustly elicit eye movements (Bruce et al., 1985). Thus, the FEF appears to be a functional intermediate between the PFC and the frontal motor areas, with characteristics analogous to the PMd. The FEF neurons show sustained activity during memory-guided saccade tasks indicative of saccade planning and working memory (Bruce and Goldberg, 1985). Mirroring the LIP, the FEF plays an important role in the control of visual attention as well, but its position in the functional hierarchy may differ from the LIP (Moore and Fallah, 2001; Wardak et al., 2006; Schall et al., 2011). More specifically, in a visual search task, the spatial attention signal guided by a top-down computation using the prior cue emerges in the FEF before the LIP, whereas the opposite temporal order occurs for bottom-up sensory driven attention (Buschman and Miller, 2007; Ibos et al., 2013). Moreover, the FEF neurons show context-dependent neural activity and dynamics in response to the same sensory stimuli, suggesting that the FEF might guide abstract rule-based action selection (Sato and Schall, 2003; Mante et al., 2013). In sum, the primate frontal areas PMd and FEF are involved in organizing goal-directed behavior including action control, rule-based action selection, and top-down control of attention.

Whether and where rodents have brain areas homologous to the primate PFC is an unsettled matter (Carlén, 2017; Laubach et al., 2018). This issue is particularly controversial for the dorsolateral PFC, which has been linked to high-level executive function in primates (Miller, 2000). The rodent frontal cortex lacks granular regions, which were originally used to define the primate PFC and receive inputs from sensorimotor areas more extensively than the primate PFC (Carlén, 2017; Laubach et al., 2018). Nevertheless, the rodent frontal regions that are often referred to as the medial PFC show some similarity to the primate medial frontal areas: the rodent medial PFC receives inputs from the mediodorsal thalamic nucleus and shows cytoarchitecturally robust subdivisions similar to the primate medial frontal areas (Schaeffer et al., 2020). Despite this similarity, however, the functional connectivity of the medial frontal areas with other brain areas diverges between the two species. Thus, it remains to be determined to what extent the rodent medial frontal areas resemble the primate medal frontal areas.

Notwithstanding the above caveat, the dorsal part of the rodent medial frontal areas includes part of a region referred to as the M2, which shows similar, albeit not identical, anatomical connections and electrophysiological properties as the primate PMd and FEF (Barthas and Kwan, 2017; Ebbesen et al., 2018). First, in rats, the M2 frontal orienting field (FOF) very closely resembles the primate FEF (Erlich et al., 2011). Intracortical microstimulation of the rat FOF leads to orienting motions (Sinnamon and Galer, 1984). Unilateral lesions or inactivation of the FOF impairs contralateral orienting responses (Cowey and Bozek, 1974; Hanks et al., 2015; Kopec et al., 2015). Furthermore, the FOF neurons have delay period activity that predicts the direction of the upcoming orienting motion in a memory-guided orientation task (Erlich et al., 2015; Kopec et al., 2015). Similar to the rat FOF, Itokazu et al. (2018) found that neurons in the mouse M2 encode the direction of upcoming saccades, and optogenetic suppression of the M2 neurons impairs contraversive saccades. Second, the rodent M2 exhibits functional similarities to the primate PMd as well; lesion of the rat M2 impairs conditional sensorimotor associations (Passingham et al., 1988), indicating that this area is also important for abstract rule-based action selection. Related to this rule-dependent action selection, the rat M2 has also been implicated in choice-outcome-dependent reinforcement learning (Sul et al., 2011). Lastly, mirroring the primate PMd and FEF, the M2 forms reciprocal connections with the PPC.

Given the functional and anatomical similarities between the rodent M2 and the primate PMd/FEF, the current review addresses the question of what information is transmitted from the frontal to the parietal areas, focusing on the rodent M2 and the primate PMd/FEF. One of the first rodent studies that directly examined this question is the aforementioned study by Itokazu et al. (2018). They recorded the axonal activity of M2 neurons projecting to the PPC in mice and found that the general population of M2 neurons encodes both the visual stimulus and movement target information in a mixed manner, similar to the general population of PPC neurons (top histogram in Figure 2). However, M2 neurons projecting to the PPC predominantly encode the movement target information (right histogram in Figure 2), in stark contrast to the PPC neurons projecting to the M2, which preferentially encode the stimulus information (left histogram in Figure 2). That is, the M2 sends motor target information to the PPC.

The explicit purpose of the motor target information flow from the M2 to the PPC has yet to be characterized, but decades of primate research offer some compelling hypotheses. Research has shown that intracortical microstimulation of the FEF in macaques activates the LIP in the PPC and visual areas, consistent with the anatomical connectivity (Ekstrom et al., 2008). Furthermore, this microstimulation could enhance the response of neurons in visual areas and the subject’s performance in attention-demanding visual search tasks, mimicking the effect of top-down attention (Noudoost et al., 2010). Analogously, mouse studies have demonstrated that top-down projection neurons from the M2 or adjacent cingulate region to primary sensory areas modulate the sensory-driven neural response and sensory perception (Zhang et al., 2014; Manita et al., 2015; Nelson and Mooney, 2016). These findings suggest that frontal areas may provide top-down signals that regulate sensory processing in a context-dependent manner.

Intriguingly, perceptual enhancement can also occur for sensory stimuli at planned movement targets, indicating a link between movement planning and attention processing (Baldauf and Deubel, 2008). Thus, one potential role of the motor target information flow from the M2 to the PPC might be related to allocating spatial attention to the planned movement target via the PPC. Although motor-target associated attention deployment has not been explicitly tested in mice, ample evidence indicates that body movement greatly modulates neuronal activity across whole brain regions and enhances stimulus responses of neurons in the primary visual cortex (Niell and Stryker, 2010; Musall et al., 2019). Moreover, neuronal responses to the same stimulus vary depending on the type of future movement (Poort et al., 2015). Thus, it seems plausible that movement plans influence sensory responses and perception in mice as well.

In addition to attention processing, the PPC has been implicated in various other functions that may utilize motor target information originating from the frontal areas (Andersen et al., 2010). Such functions in primates include the spatial control of movement, hand-eye coordination to see where to reach, working memory to preserve motor target information during a delay period, and forward models to predict the sensory state of body parts using efference copy and sensory feedback for efficient feedback control (Mulliken et al., 2008; Hwang et al., 2012, 2014; Hart and Huk, 2020). Given the variety of potential functions, frontoparietal connectivity that relays the top-down motor target information may also be organized in multiple parallel pathways, each serving different functions such as attention, multi-body part coordination, and forward models (downward arrows in Figure 2). Testing this hypothesis will require establishing behavioral paradigms to characterize each of these spatial functions in mice and examining the information flow from the M2 to the PPC during behavioral tasks.

## Canonical Information Flows in the Frontoparietal Network

Based on the recently identified pathway-specific information flows in mice and extensively characterized neural response properties in primates, we propose the following canonical organization of information flows in the frontoparietal network (Figure 2). The parietofrontal pathway relays bottom-up target information for sensory-driven reflexive movement, while the frontoparietal pathway relays top-down target information that is cognitive rule-based. The reflexive motor plan originating from the parietofrontal pathway may be endorsed or suppressed in the frontal areas that compute proper motor plans based on abstract, contextual rules. The endorsed reflexive or alternative motor plan in the frontal areas drives motor programs in the frontal motor areas. This frontal motor plan is also sent to the PPC so that it may be used for other functions requiring motor plan information such as attention, multi-body part coordination, and forward models (Andersen et al., 2010). Furthermore, the motor plan might be maintained as working memory through the reciprocal recurrent connectivity between the PPC and frontal areas for delayed execution (Salazar et al., 2012; Murray et al., 2017; Hart and Huk, 2020). Lastly, internally generated preferred motor plans in the PPC may also take advantage of the parietofrontal pathway to prime the frontal motor circuit to initiate motor programs for preferred movements (Hwang et al., 2019). This canonical computational architecture presents a number of testable hypotheses described in earlier sections, which can further delineate the circuit mechanisms and information processing in the frontoparietal network for a wide range of cognitive functions underlying goal-directed behavior.

## Frontoparietal Network in Alzheimer’s Disease

Dysfunction of the frontoparietal network could underlie many neurological/neuropsychological diseases including Alzheimer’s disease (AD). The role of the frontoparietal network has been underappreciated in AD; traditionally, AD pathology has been characterized as neurodegeneration in the medial temporal structures (e.g., hippocampus, entorhinal cortex) and memory impairments have been the most representative AD symptoms. However, recently AD symptoms in sensory and motor systems began to gain more and more attention (Albers et al., 2015). Two lines of evidence below suggest that alterations in the frontoparietal network may play crucial roles in AD symptomology, and we propose that studying the long-range connections in this network in AD mouse model is an attractive future direction.

First, the neuropathology of AD (i.e., deposition of amyloid β peptide and neurofibrillary tangles) is frequently observed in the sensory and frontal areas at an early disease phase prior to cognitive decline (Thal et al., 2002; McKee et al., 2006; Palmqvist et al., 2017), in some cases earlier than medial temporal structures. Similarly, AD patients in early phases of AD exhibit deficits in key cognitive functions that involve the frontoparietal network such as attention (Perry and Hodges, 1999), motor planning (Manckoundia et al., 2006), and sensorimotor behaviors (Albers et al., 2015).

Second, AD is increasingly viewed as a disconnection syndrome, where the long-range cortical interactions are impaired (Delbeuck et al., 2003). The evidence supporting this view derives from human fMRI studies demonstrating a reduction in functional connectivity in the frontoparietal network in AD patients (Greicius et al., 2004; Sorg et al., 2007; Zhao et al., 2018). Reduced functional connectivity has been reported even in prodromal-stage AD patients who exhibit amyloid β deposition without experiencing cognitive deficits (Hedden et al., 2009; Stam et al., 2009; Sanz-Arigita et al., 2010; Sheline et al., 2010a, b; Brier et al., 2012; Sheline and Raichle, 2013; Palmqvist et al., 2017), and has been replicated in AD model mice (Bero et al., 2012; Busche et al., 2015). The reduced functional connectivity is such a reliable and general feature of AD that it has been proposed as a biomarker for the disease (Balthazar et al., 2014).

It is reasonable to speculate that the reduced functional connectivity in AD patients might reflect impaired long-range connectivity (Honey et al., 2007; Bressler and Menon, 2010). Yet, the functional connectivity in AD patients so far has been inferred only from temporal correlations of fMRI signals that reflect the complex, collective metabolic activity of thousands of neurons including both long-range projection and local neurons (Logothetis et al., 2001; Glover, 2011). Furthermore, altered temporal correlations could arise from changes in indirect anatomical connections between cortical areas, whether or not their direct connections are altered. Thus, the circuit mechanisms underlying the observed functional connectivity in AD patients remain to be revealed. Future work that combines *in vivo* imaging and cell-type-specific labeling of monosynaptic long-range inputs, as discussed above, will provide key information regarding the relationship between reduced functional connectivity and the cognitive and goal-directed behavior deficits in AD.

## Discussion

The frontoparietal network might have evolved from the pressure to support goal-directed behavior, allowing context-dependent flexible association and temporal separation between sensory stimuli and motor responses. The parietofrontal pathway transmits bottom-up sensory-driven information to drive automatic or default responses similar to reflexes. The bottom-up responses can be enforced, temporarily withheld, or canceled in context-dependent manners through interaction with the frontal areas specialized for the computation of cognitive rule-based responses. The frontoparietal pathway transmits cognitively processed responses so that it can be used by the parietal areas specialized for spatial information processing for attention allocation, multi-body part coordination, and forward models. We hope this canonical scheme of parallel information flows in the frontoparietal network provides holistic insights into the dynamic information processing in this network and guides future research delineating the computational hierarchy and supporting circuit mechanisms in a systematic way. Furthermore, a detailed understanding of the frontoparietal network can shed light on the neural basis of impaired goal-directed behavior in various neurological and psychiatric disorders including AD.

## Author Contributions

EH, TRS, and TKS: conceptualization and writing. TRS and TKS: funding acquisition. All authors contributed to the article and approved the submitted version.

## Conflict of Interest

The authors declare that the research was conducted in the absence of any commercial or financial relationships that could be construed as a potential conflict of interest.

## Publisher’s Note

All claims expressed in this article are solely those of the authors and do not necessarily represent those of their affiliated organizations, or those of the publisher, the editors and the reviewers. Any product that may be evaluated in this article, or claim that may be made by its manufacturer, is not guaranteed or endorsed by the publisher.

## References

[B1] Akrami A. Kopec C. D. Diamond M. E. Brody C. D. (2018). Posterior parietal cortex represents sensory history and mediates its effects on behaviour. *Nature* 554:368. 10.1038/nature25510 29414944

[B2] Albers M. W. Gilmore G. C. Kaye J. Murphy C. Wingfield A. Bennett D. A. (2015). At the interface of sensory and motor dysfunctions and Alzheimer’s disease. *Alzheimers Dement* 11 70–98. 25022540 10.1016/j.jalz.2014.04.514 PMC4287457

[B3] Andersen R. A. Andersen K. N. Hwang E. J. Hauschild M. (2014). Optic ataxia: from Balint’s syndrome to the parietal reach region. *Neuron* 81 967–983. 10.1016/j.neuron.2014.02.025 24607223 PMC4000741

[B4] Andersen R. A. Asanuma C. Essick G. Siegel R. M. (1990). Corticocortical connections of anatomically and physiologically defined subdivisions within the inferior parietal lobule. *J. Comp. Neurol.* 296 65–113. 10.1002/cne.902960106 2358530

[B5] Andersen R. A. Buneo C. A. (2002). Intentional maps in posterior parietal cortex. *Annu. Rev. Neurosci.* 25 189–220. 10.1146/annurev.neuro.25.112701.142922 12052908

[B6] Andersen R. A. Cui H. (2009). Intention, action planning, and decision making in parietal-frontal circuits. *Neuron* 63 568–583. 10.1016/j.neuron.2009.08.028 19755101

[B7] Andersen R. A. Hwang E. J. Mulliken G. H. (2010). Cognitive neural prosthetics. *Annu. Rev. Psychol.* 61 169–190. 19575625 10.1146/annurev.psych.093008.100503 PMC2849803

[B8] Baldauf D. Deubel H. (2008). Visual attention during the preparation of bimanual movements. *Vision Res.* 48 549–563. 10.1016/j.visres.2007.11.023 18206205

[B9] Balthazar M. L. de Campos B. M. Franco A. R. Damasceno B. P. Cendes F. (2014). Whole cortical and default mode network mean functional connectivity as potential biomarkers for mild Alzheimer’s disease. *Psychiatry Res.* 221 37–42. 10.1016/j.pscychresns.2013.10.010 24268581

[B10] Barthas F. Kwan A. C. (2017). Secondary motor cortex: where ‘Sensory’ Meets ‘Motor’ in the rodent frontal cortex. *Trends Neurosci.* 40 181–193. 10.1016/j.tins.2016.11.006 28012708 PMC5339050

[B11] Battaglia-Mayer A. Caminiti R. (2019). Corticocortical systems underlying high-order motor control. *J. Neurosci.* 39 4404–4421. 10.1523/jneurosci.2094-18.2019 30886016 PMC6554627

[B12] Bero A. W. Bauer A. Q. Stewart F. R. White B. R. Cirrito J. R. Raichle M. E. (2012). Bidirectional relationship between functional connectivity and amyloid-beta deposition in mouse brain. *J. Neurosci.* 32 4334–4340. 10.1523/jneurosci.5845-11.2012 22457485 PMC3326343

[B13] Bishop P. O. Burke W. Davis R. (1962). Single-unit recording from antidromically activated optic radiation neurones. *J. Physiol.* 162 432–450. 10.1113/jphysiol.1962.sp006943 13869505 PMC1359668

[B14] Bisley J. W. Goldberg M. E. (2003). Neuronal activity in the lateral intraparietal area and spatial attention. *Science* 299 81–86. 10.1126/science.1077395 12511644

[B15] Bressler S. L. Menon V. (2010). Large-scale brain networks in cognition: emerging methods and principles. *Trends Cogn. Sci.* 14 277–290. 10.1016/j.tics.2010.04.004 20493761

[B16] Brier M. R. Thomas J. B. Snyder A. Z. Benzinger T. L. Zhang D. Raichle M. E. (2012). Loss of intranetwork and internetwork resting state functional connections with Alzheimer’s disease progression. *J. Neurosci.* 32 8890–8899. 10.1523/jneurosci.5698-11.2012 22745490 PMC3458508

[B17] Bruce C. J. Goldberg M. E. (1985). Primate frontal eye fields. I. Single neurons discharging before saccades. *J. Neurophysiol.* 53 603–635. 10.1152/jn.1985.53.3.603 3981231

[B18] Bruce C. J. Goldberg M. E. Bushnell M. C. Stanton G. B. (1985). Primate frontal eye fields. II. Physiological and anatomical correlates of electrically evoked eye movements. *J. Neurophysiol.* 54 714–734. 10.1152/jn.1985.54.3.714 4045546

[B19] Busche M. A. Kekus M. Adelsberger H. Noda T. Forstl H. Nelken I. (2015). Rescue of long-range circuit dysfunction in Alzheimer’s disease models. *Nat. Neurosci.* 18 1623–1630. 10.1038/nn.4137 26457554

[B20] Buschman T. J. Miller E. K. (2007). Top-down versus bottom-up control of attention in the prefrontal and posterior parietal cortices. *Science* 315 1860–1862. 10.1126/science.1138071 17395832

[B21] Carlén M. (2017). What constitutes the prefrontal cortex? *Science* 358 478–482. 10.1126/science.aan8868 29074767

[B22] Cavada C. Goldman-Rakic P. S. (1989). Posterior parietal cortex in rhesus monkey: II. Evidence for segregated corticocortical networks linking sensory and limbic areas with the frontal lobe. *J. Comp. Neurol.* 287 422–445. 10.1002/cne.902870403 2477406

[B23] Chen J. L. Carta S. Soldado-Magraner J. Schneider B. L. Helmchen F. (2013). Behaviour-dependent recruitment of long-range projection neurons in somatosensory cortex. *Nature* 499 336–340. 10.1038/nature12236 23792559

[B24] Christopoulos V. N. Bonaiuto J. Kagan I. Andersen R. A. (2015). Inactivation of parietal reach region affects reaching but not saccade choices in internally guided decisions. *J. Neurosci.* 35 11719–11728. 10.1523/jneurosci.1068-15.2015 26290248 PMC4540805

[B25] Churchland M. M. Santhanam G. Shenoy K. V. (2006a). Preparatory activity in premotor and motor cortex reflects the speed of the upcoming reach. *J. Neurophysiol.* 96 3130–3146. 10.1152/jn.00307.2006 16855111

[B26] Churchland M. M. Yu B. M. Ryu S. I. Santhanam G. Shenoy K. V. (2006b). Neural variability in premotor cortex provides a signature of motor preparation. *J. Neurosci.* 26 3697–3712. 10.1523/jneurosci.3762-05.2006 16597724 PMC6674116

[B27] Churchland M. M. Shenoy K. V. (2007). Delay of movement caused by disruption of cortical preparatory activity. *J. Neurophysiol.* 97 348–359. 10.1152/jn.00808.2006 17005608

[B28] Cisek P. Crammond D. J. Kalaska J. F. (2003). Neural activity in primary motor and dorsal premotor cortex in reaching tasks with the contralateral versus ipsilateral arm. *J. Neurophysiol.* 89 922–942. 10.1152/jn.00607.2002 12574469

[B29] Condylis C. Lowet E. Ni J. Bistrong K. Ouellette T. Josephs N. (2020). Context-dependent sensory processing across primary and secondary somatosensory cortex. *Neuron* 106 515.e5–525.e5. 32164873 10.1016/j.neuron.2020.02.004 PMC7210055

[B30] Copits B. A. Gowrishankar R. O’Neill P. R. Li J. N. Girven K. S. Yoo J. J. (2021). A photoswitchable GPCR-based opsin for presynaptic inhibition. *Neuron* 109 1791.e11–1809.e11. 33979635 10.1016/j.neuron.2021.04.026 PMC8194251

[B31] Corbetta M. (1998). Frontoparietal cortical networks for directing attention and the eye to visual locations: identical, independent, or overlapping neural systems? *Proc. Natl. Acad. Sci. U.S.A.* 95 831–838. 10.1073/pnas.95.3.831 9448248 PMC33805

[B32] Cowey A. Bozek T. (1974). Contralateral “neglect” after unilateral dorsomedial prefrontal lesions in rats. *Brain Res.* 72 53–63. 10.1016/0006-8993(74)90649-0 4830476

[B33] Crammond D. J. Kalaska J. F. (1994). Modulation of preparatory neuronal activity in dorsal premotor cortex due to stimulus-response compatibility. *J. Neurophysiol.* 71 1281–1284. 10.1152/jn.1994.71.3.1281 8201421

[B34] Delbeuck X. Van der Linden M. Collette F. (2003). Alzheimer’s disease as a disconnection syndrome? *Neuropsychol. Rev.* 13 79–92. 12887040 10.1023/a:1023832305702

[B35] Dorris M. C. Glimcher P. W. (2004). Activity in posterior parietal cortex is correlated with the relative subjective desirability of action. *Neuron* 44 365–378. 10.1016/j.neuron.2004.09.009 15473973

[B36] Driscoll L. N. Pettit N. L. Minderer M. Chettih S. N. Harvey C. D. (2017). Dynamic reorganization of neuronal activity patterns in parietal cortex. *Cell* 170 986.e16–999.e16. 28823559 10.1016/j.cell.2017.07.021 PMC5718200

[B37] Ebbesen C. L. Insanally M. N. Kopec C. D. Murakami M. Saiki A. Erlich J. C. (2018). More than Just a “Motor”: recent surprises from the frontal cortex. *J. Neurosci.* 38 9402–9413. 10.1523/jneurosci.1671-18.2018 30381432 PMC6209835

[B38] Economo M. N. Viswanathan S. Tasic B. Bas E. Winnubst J. Menon V. (2018). Distinct descending motor cortex pathways and their roles in movement. *Nature* 563:79. 10.1038/s41586-018-0642-9 30382200

[B39] Ekstrom L. B. Roelfsema P. R. Arsenault J. T. Bonmassar G. Vanduffel W. (2008). Bottom-up dependent gating of frontal signals in early visual cortex. *Science* 321 414–417. 10.1126/science.1153276 18635806 PMC3011100

[B40] Erlich J. C. Bialek M. Brody C. D. (2011). A cortical substrate for memory-guided orienting in the rat. *Neuron* 72 330–343. 10.1016/j.neuron.2011.07.010 22017991 PMC3212026

[B41] Erlich J. C. Brunton B. W. Duan C. A. Hanks T. D. Brody C. D. (2015). Distinct effects of prefrontal and parietal cortex inactivations on an accumulation of evidence task in the rat. *eLife Sci.* 4:e05457. 10.7554/eLife.05457 PMC4392479 25869470

[B42] Felleman D. J. Van Essen D. C. (1991). Distributed hierarchical processing in the primate cerebral cortex. *Cereb. Cortex* 1 1–47. 10.1093/cercor/1.1.1 1822724

[B43] Ferraina S. Paré M. Wurtz R. H. (2002). Comparison of cortico-cortical and cortico-collicular signals for the generation of saccadic eye movements. *J. Neurophysiol.* 87 845–858. 10.1152/jn.00317.2001 11826051

[B44] Gail A. Klaes C. Westendorff S. (2009). Implementation of spatial transformation rules for goal-directed reaching via gain modulation in monkey parietal and premotor cortex. *J. Neurosci.* 29 9490–9499. 10.1523/jneurosci.1095-09.2009 19641112 PMC6666548

[B45] Glickfeld L. L. Andermann M. L. Bonin V. Reid R. C. (2013). Cortico-cortical projections in mouse visual cortex are functionally target specific. *Nat. Neurosci.* 16 219–226. 10.1038/nn.3300 23292681 PMC3808876

[B46] Glimcher P. W. (2003). The neurobiology of visual-saccadic decision making. *Annu. Rev. Neurosci.* 26 133–179. 14527268 10.1146/annurev.neuro.26.010302.081134

[B47] Glover G. H. (2011). Overview of functional magnetic resonance imaging. *Neurosurg. Clin. N. Am.* 22 133–139. 21435566 10.1016/j.nec.2010.11.001 PMC3073717

[B48] Gnadt J. W. Andersen R. A. (1988). Memory related motor planning activity in posterior parietal cortex of macaque. *Exp. Brain Res.* 70 216–220. 3402565 10.1007/BF00271862

[B49] Goard M. J. Pho G. N. Woodson J. Sur M. (2016). Distinct roles of visual, parietal, and frontal motor cortices in memory-guided sensorimotor decisions. *eLife* 5:e13764. 10.7554/eLife.13764 PMC4974053 27490481

[B50] Gradinaru V. Thompson K. R. Zhang F. Mogri M. Kay K. Schneider M. B. (2007). Targeting and readout strategies for fast optical neural control in vitro and in vivo. *J. Neurosci.* 27 14231–14238. 10.1523/jneurosci.3578-07.2007 18160630 PMC6673457

[B51] Greicius M. D. Srivastava G. Reiss A. L. Menon V. (2004). Default-mode network activity distinguishes Alzheimer’s disease from healthy aging: evidence from functional MRI. *Proc. Natl. Acad. Sci. U.S.A.* 101 4637–4642. 10.1073/pnas.0308627101 15070770 PMC384799

[B52] Guo Z. V. Li N. Huber D. Ophir E. Gutnisky D. Ting J. T. (2014). Flow of cortical activity underlying a tactile decision in mice. *Neuron* 81 179–194. 10.1016/j.neuron.2013.10.020 24361077 PMC3984938

[B53] Hanks T. D. Kopec C. D. Brunton B. W. Duan C. A. Erlich J. C. Brody C. D. (2015). Distinct relationships of parietal and prefrontal cortices to evidence accumulation. *Nature* 520 220–223. 10.1038/nature14066 25600270 PMC4835184

[B54] Hart E. Huk A. C. (2020). Recurrent circuit dynamics underlie persistent activity in the macaque frontoparietal network. *eLife* 9:e52460. 10.7554/eLife.52460 PMC7205463 32379044

[B55] Harvey C. D. Coen P. Tank D. W. (2012). Choice-specific sequences in parietal cortex during a virtual-navigation decision task. *Nature* 484 62–68. 10.1038/nature10918 22419153 PMC3321074

[B56] Hedden T. Van Dijk K. R. Becker J. A. Mehta A. Sperling R. A. Johnson K. A. (2009). Disruption of functional connectivity in clinically normal older adults harboring amyloid burden. *J. Neurosci.* 29 12686–12694. 10.1523/jneurosci.3189-09.2009 19812343 PMC2808119

[B57] Honey C. J. Kotter R. Breakspear M. Sporns O. (2007). Network structure of cerebral cortex shapes functional connectivity on multiple time scales. *Proc. Natl. Acad. Sci. U.S.A.* 104 10240–10245. 10.1073/pnas.0701519104 17548818 PMC1891224

[B58] Hovde K. Gianatti M. Witter M. P. Whitlock J. R. (2018). Architecture and organization of mouse posterior parietal cortex relative to extrastriate areas. *Eur. J. Neurosci.* 49 1313–1329. 30456892 10.1111/ejn.14280

[B59] Hwang E. J. Dahlen J. E. Mukundan M. Komiyama T. (2017). History-based action selection bias in posterior parietal cortex. *Nat. Commun.* 8:1242. 10.1038/s41467-017-01356-z PMC5663966 29089500

[B60] Hwang E. J. Hauschild M. Wilke M. Andersen R. A. (2012). Inactivation of the parietal reach region causes optic ataxia, impairing reaches but not saccades. *Neuron* 76 1021–1029. 10.1016/j.neuron.2012.10.030 23217749 PMC3597097

[B61] Hwang E. J. Hauschild M. Wilke M. Andersen R. A. (2014). Spatial and temporal eye–hand coordination relies on the parietal reach region. *J. Neurosci.* 34 12884–12892. 10.1523/jneurosci.3719-13.2014 25232123 PMC4166167

[B62] Hwang E. J. Link T. D. Hu Y. Y. Lu S. Wang E. H.-J. Lilascharoen V. (2019). Corticostriatal flow of action selection bias. *Neuron* 104 1126.e6–1140.e6. 31706697 10.1016/j.neuron.2019.09.028 PMC6923603

[B63] Ibos G. Duhamel J.-R. Ben Hamed S. (2013). A functional hierarchy within the parietofrontal network in stimulus selection and attention control. *J. Neurosci.* 33 8359–8369. 10.1523/jneurosci.4058-12.2013 23658175 PMC6619613

[B64] Itokazu T. Hasegawa M. Kimura R. Osaki H. Albrecht U.-R. Sohya K. (2018). Streamlined sensory motor communication through cortical reciprocal connectivity in a visually guided eye movement task. *Nat. Commun.* 9:338. 10.1038/s41467-017-02501-4 PMC5780522 29362373

[B65] Jarosiewicz B. Schummers J. Malik W. Q. Brown E. N. Sur M. (2012). Functional biases in visual cortex neurons with identified projections to higher cortical targets. *Curr. Biol.* 22 269–277. 10.1016/j.cub.2012.01.011 22305753 PMC3288404

[B66] Johnson P. B. Ferraina S. Bianchi L. Caminiti R. (1996). Cortical networks for visual reaching: physiological and anatomical organization of frontal and parietal lobe arm regions. *Cereb. Cortex* 6 102–119. 10.1093/cercor/6.2.102 8670643

[B67] Katz L. N. Yates J. L. Pillow J. W. Huk A. C. (2016). Dissociated functional significance of decision-related activity in the primate dorsal stream. *Nature* 535 285–288. 10.1038/nature18617 27376476 PMC4966283

[B68] Kopec C. D. Erlich J. C. Brunton B. W. Deisseroth K. Brody C. D. (2015). Cortical and subcortical contributions to short-term memory for orienting movements. *Neuron* 88 367–377. 10.1016/j.neuron.2015.08.033 26439529 PMC5521275

[B69] Kurata K. Hoffman D. S. (1994). Differential effects of muscimol microinjection into dorsal and ventral aspects of the premotor cortex of monkeys. *J. Neurophysiol.* 71 1151–1164. 10.1152/jn.1994.71.3.1151 8201409

[B70] Kwon S. E. Yang H. Minamisawa G. O’Connor D. H. (2016). Sensory and decision-related activity propagate in a cortical feedback loop during touch perception. *Nat. Neurosci.* 19 1243–1249. 10.1038/nn.4356 27437910 PMC5003632

[B71] Laubach M. Amarante L. M. Swanson K. White S. R. (2018). What, if anything, is rodent prefrontal cortex? *eNeuro* 5:ENEURO.315–ENEURO.318. 10.1523/ENEURO.0315-18.2018 PMC6220587 30406193

[B72] Licata A. M. Kaufman M. T. Raposo D. Ryan M. B. Sheppard J. P. Churchland A. K. (2017). Posterior parietal cortex guides visual decisions in rats. *J. Neurosci.* 37 4954–4966. 10.1523/jneurosci.0105-17.2017 28408414 PMC5426183

[B73] Lima S. Q. Hromadka T. Znamenskiy P. Zador A. M. (2009). PINP: a new method of tagging neuronal populations for identification during in vivo electrophysiological recording. *PLoS One* 4:e6099. 10.1371/journal.pone.0006099 19584920 PMC2702752

[B74] Logothetis N. K. Pauls J. Augath M. Trinath T. Oeltermann A. (2001). Neurophysiological investigation of the basis of the fMRI signal. *Nature* 412 150–157. 10.1038/35084005 11449264

[B75] Lyamzin D. Benucci A. (2019). The mouse posterior parietal cortex: anatomy and functions. *Neurosci. Res.* 140 14–22. 10.1016/j.neures.2018.10.008 30465783

[B76] Mahn M. Prigge M. Ron S. Levy R. Yizhar O. (2016). Biophysical constraints of optogenetic inhibition at presynaptic terminals. *Nat. Neurosci.* 19 554–556. 10.1038/nn.4266 26950004 PMC4926958

[B77] Mahn M. Saraf-Sinik I. Patil P. Pulin M. Bitton E. Karalis N. (2021). Efficient optogenetic silencing of neurotransmitter release with a mosquito rhodopsin. *Neuron* 109 1621.e8–1635.e8. 33979634 10.1016/j.neuron.2021.03.013 PMC7611984

[B78] Manckoundia P. Mourey F. Pfitzenmeyer P. Papaxanthis C. (2006). Comparison of motor strategies in sit-to-stand and back-to-sit motions between healthy and Alzheimer’s disease elderly subjects. *Neuroscience* 137 385–392. 10.1016/j.neuroscience.2005.08.079 16289889

[B79] Manita S. Suzuki T. Homma C. Matsumoto T. Odagawa M. Yamada K. (2015). A top-down cortical circuit for accurate sensory perception. *Neuron* 86 1304–1316. 10.1016/j.neuron.2015.05.006 26004915

[B80] Mante V. Sussillo D. Shenoy K. V. Newsome W. T. (2013). Context-dependent computation by recurrent dynamics in prefrontal cortex. *Nature* 503 78–84. 10.1038/nature12742 24201281 PMC4121670

[B81] McKee A. C. Au R. Cabral H. J. Kowall N. W. Seshadri S. Kubilus C. A. (2006). Visual association pathology in preclinical Alzheimer disease. *J. Neuropathol. Exp. Neurol.* 65 621–630. 10.1097/00005072-200606000-00010 16783172

[B82] Miller E. K. (2000). The prefontral cortex and cognitive control. *Nat. Rev. Neurosci.* 1 59–65. 10.1038/35036228 11252769

[B83] Mitz A. R. Godschalk M. Wise S. P. (1991). Learning-dependent neuronal activity in the premotor cortex: activity during the acquisition of conditional motor associations. *J. Neurosci.* 11 1855–1872. 10.1523/jneurosci.11-06-01855.1991 2045890 PMC6575410

[B84] Moore T. Fallah M. (2001). Control of eye movements and spatial attention. *Proc. Natl. Acad. Sci. U.S.A.* 98 1273–1276. 11158629 10.1073/pnas.021549498 PMC14744

[B85] Morcos A. S. Harvey C. D. (2016). History-dependent variability in population dynamics during evidence accumulation in cortex. *Nat. Neurosci.* 19 1672–1681. 10.1038/nn.4403 27694990 PMC5127723

[B86] Mulliken G. H. Musallam S. Andersen R. A. (2008). Forward estimation of movement state in posterior parietal cortex. *Proc. Natl. Acad. Sci. U.S.A.* 105 8170–8177. 10.1073/pnas.0802602105 18499800 PMC2448809

[B87] Murray J. D. Jaramillo J. Wang X.-J. (2017). Working memory and decision-making in a frontoparietal circuit model. *J. Neurosci.* 37 12167–12186. 10.1523/jneurosci.0343-17.2017 29114071 PMC5729190

[B88] Musall S. Kaufman M. T. Juavinett A. L. Gluf S. Churchland A. K. (2019). Single-trial neural dynamics are dominated by richly varied movements. *Nat. Neurosci.* 22 1677–1686. 10.1038/s41593-019-0502-4 31551604 PMC6768091

[B89] Musallam S. Corneil B. D. Greger B. Scherberger H. Andersen R. A. (2004). Cognitive control signals for neural prosthetics. *Science* 305 258–262. 10.1126/science.1097938 15247483

[B90] Nelson A. Mooney R. (2016). The basal forebrain and motor cortex provide convergent yet distinct movement-related inputs to the auditory cortex. *Neuron* 90 635–648. 10.1016/j.neuron.2016.03.031 27112494 PMC4866808

[B91] Niell C. M. Stryker M. P. (2010). Modulation of visual responses by behavioral state in mouse visual cortex. *Neuron* 65 472–479. 10.1016/j.neuron.2010.01.033 20188652 PMC3184003

[B92] Noudoost B. Chang M. H. Steinmetz N. A. Moore T. (2010). Top-down control of visual attention. *Curr. Opin. Neurobiol.* 20 183–190. 20303256 10.1016/j.conb.2010.02.003 PMC2901796

[B93] Olsen G. M. Hovde K. Kondo H. Sakshaug T. Sømme H. H. Whitlock J. R. (2019). Organization of posterior parietal–frontal connections in the rat. *Front. Syst. Neurosci.* 13:38. 10.3389/fnsys.2019.00038 31496940 PMC6713060

[B94] Palmqvist S. Scholl M. Strandberg O. Mattsson N. Stomrud E. Zetterberg H. (2017). Earliest accumulation of beta-amyloid occurs within the default-mode network and concurrently affects brain connectivity. *Nat. Commun.* 8:1214. 10.1038/s41467-017-01150-x PMC5663717 29089479

[B95] Passingham R. E. (1988). Premotor cortex and preparation for movement. *Exp. Brain Res.* 70 590–596. 3384057 10.1007/BF00247607

[B96] Passingham R. E. Myers C. Rawlins N. Lightfoot V. Fearn S. (1988). Premotor cortex in the rat. *Behav. Neurosci.* 102 101–109. 3355650 10.1037//0735-7044.102.1.101

[B97] Perry R. J. Hodges J. R. (1999). Attention and executive deficits in Alzheimer’s disease. A critical review. *Brain* 122(Pt 3) 383–404. 10.1093/brain/122.3.383 10094249

[B98] Pesaran B. Nelson M. J. Andersen R. A. (2008). Free choice activates a decision circuit between frontal and parietal cortex. *Nature* 453 406–409. 10.1038/nature06849 18418380 PMC2728060

[B99] Petreanu L. Gutnisky D. A. Huber D. Xu N. O’Connor D. H. Tian L. (2012). Activity in motor-sensory projections reveals distributed coding in somatosensation. *Nature* 489 299–303. 10.1038/nature11321 22922646 PMC3443316

[B100] Platt M. L. Glimcher P. W. (1999). Neural correlates of decision variables in parietal cortex. *Nature* 400 233–238. 10.1038/22268 10421364

[B101] Poort J. Khan A. G. Pachitariu M. Nemri A. Orsolic I. Krupic J. (2015). Learning enhances sensory and multiple non-sensory representations in primary visual cortex. *Neuron* 86 1478–1490. 10.1016/j.neuron.2015.05.037 26051421 PMC4503798

[B102] Ptak R. Schnider A. Fellrath J. (2017). The dorsal frontoparietal network: a core system for emulated action. *Trends Cogn. Sci.* 21 589–599. 10.1016/j.tics.2017.05.002 28578977

[B103] Raposo D. Kaufman M. T. Churchland A. K. (2014). A category-free neural population supports evolving demands during decision-making. *Nat. Neurosci.* 17 1784–1792. 10.1038/nn.3865 25383902 PMC4294797

[B104] Reardon T. R. Murray A. J. Turi G. F. Wirblich C. Croce K. R. Schnell M. J. (2016). Rabies virus CVS-N2cΔG strain enhances retrograde synaptic transfer and neuronal viability. *Neuron* 89 711–724. 10.1016/j.neuron.2016.01.004 26804990 PMC4760870

[B105] Salazar R. F. Dotson N. M. Bressler S. L. Gray C. M. (2012). Content-specific fronto-parietal synchronization during visual working memory. *Science* 338 1097–1100. 10.1126/science.1224000 23118014 PMC4038369

[B106] Sanz-Arigita E. J. Schoonheim M. M. Damoiseaux J. S. Rombouts S. A. Maris E. Barkhof F. (2010). Loss of “small-world” networks in Alzheimer’s disease: graph analysis of FMRI resting-state functional connectivity. *PLoS One* 5:e13788. 10.1371/journal.pone.0013788 21072180 PMC2967467

[B107] Sato T. K. Hausser M. Carandini M. (2014). Distal connectivity causes summation and division across mouse visual cortex. *Nat. Neurosci.* 17 30–32. 10.1038/nn.3585 24241394 PMC5953407

[B108] Sato T. R. Schall J. D. (2003). Effects of stimulus-response compatibility on neural selection in frontal eye field. *Neuron* 38 637–648. 10.1016/s0896-6273(03)00237-x 12765614

[B109] Sato T. R. Svoboda K. (2010). The functional properties of barrel cortex neurons projecting to the primary motor cortex. *J. Neurosci.* 30 4256–4260. 10.1523/jneurosci.3774-09.2010 20335461 PMC6634518

[B110] Schaeffer D. J. Hori Y. Gilbert K. M. Gati J. S. Menon R. S. Everling S. (2020). Divergence of rodent and primate medial frontal cortex functional connectivity. *Proc. Natl. Acad. Sci. U.S.A.* 117 21681–21689. 10.1073/pnas.2003181117 32817555 PMC7474619

[B111] Schall J. D. Purcell B. A. Heitz R. P. Logan G. D. Palmeri T. J. (2011). Neural mechanisms of saccade target selection: gated accumulator model of the visual–motor cascade. *Eur. J. Neurosci.* 33 1991–2002. 10.1111/j.1460-9568.2011.07715.x 21645095 PMC3111938

[B112] Schwarz L. A. Miyamichi K. Gao X. J. Beier K. T. Weissbourd B. DeLoach K. E. (2015). Viral-genetic tracing of the input–output organization of a central noradrenaline circuit. *Nature* 524 88–92. 10.1038/nature14600 26131933 PMC4587569

[B113] Scott B. B. Constantinople C. M. Akrami A. Hanks T. D. Brody C. D. Tank D. W. (2017). Fronto-parietal cortical circuits encode accumulated evidence with a diversity of timescales. *Neuron* 95 385.e5–398.e5. 28669543 10.1016/j.neuron.2017.06.013 PMC9453285

[B114] Seo H. Barraclough D. J. Lee D. (2009). Lateral intraparietal cortex and reinforcement learning during a mixed-strategy game. *J. Neurosci.* 29 7278–7289. 10.1523/jneurosci.1479-09.2009 19494150 PMC2743508

[B115] Shadlen M. N. Newsome W. T. (2001). Neural basis of a perceptual decision in the parietal cortex (Area LIP) of the Rhesus Monkey. *J. Neurophysiol.* 86 1916–1936. 10.1152/jn.2001.86.4.1916 11600651

[B116] Sheline Y. I. Morris J. C. Snyder A. Z. Price J. L. Yan Z. D’Angelo G. (2010a). APOE4 allele disrupts resting state fMRI connectivity in the absence of amyloid plaques or decreased CSF Abeta42. *J. Neurosci.* 30 17035–17040. 10.1523/jneurosci.3987-10.2010 21159973 PMC3023180

[B117] Sheline Y. I. Raichle M. E. Snyder A. Z. Morris J. C. Head D. Wang S. (2010b). Amyloid plaques disrupt resting state default mode network connectivity in cognitively normal elderly. *Biol. Psychiatry* 67 584–587. 10.1016/j.biopsych.2009.08.024 19833321 PMC2829379

[B118] Sheline Y. I. Raichle M. E. (2013). Resting state functional connectivity in preclinical Alzheimer’s disease. *Biol. Psychiatry* 74 340–347. 10.1016/j.biopsych.2012.11.028 23290495 PMC3537262

[B119] Sinnamon H. M. Galer B. S. (1984). Head movements elicited by electrical stimulation of the anteromedial cortex of the rat. *Physiol. Behav.* 33 185–190. 10.1016/0031-9384(84)90098-2 6505061

[B120] Snyder L. H. Batista A. P. Andersen R. A. (1997). Coding of intention in the posterior parietal cortex. *Nature* 386 167–170. 10.1038/386167a0 9062187

[B121] Sommer M. A. Wurtz R. H. (2002). A pathway in primate brain for internal monitoring of movements. *Science* 296 1480–1482. 10.1126/science.1069590 12029137

[B122] Sorg C. Riedl V. Muhlau M. Calhoun V. D. Eichele T. Laer L. (2007). Selective changes of resting-state networks in individuals at risk for Alzheimer’s disease. *Proc. Natl. Acad. Sci. U.S.A.* 104 18760–18765. 10.1073/pnas.0708803104 18003904 PMC2141850

[B123] Sreenivasan V. Kyriakatos A. Mateo C. Jaeger D. Petersen C. C. H. (2016). Parallel pathways from whisker and visual sensory cortices to distinct frontal regions of mouse neocortex. *NPh* 4:031203. 10.1117/1.NPh.4.3.031203 PMC5120210 27921067

[B124] Stachniak T. J. Ghosh A. Sternson S. M. (2014). Chemogenetic synaptic silencing of neural circuits localizes a hypothalamus–>midbrain pathway for feeding behavior. *Neuron* 82 797–808. 10.1016/j.neuron.2014.04.008 24768300 PMC4306349

[B125] Stam C. J. de Haan W. Daffertshofer A. Jones B. F. Manshanden I. van Cappellen van Walsum A. M. (2009). Graph theoretical analysis of magnetoencephalographic functional connectivity in Alzheimer’s disease. *Brain* 132 213–224. 10.1093/brain/awn262 18952674

[B126] Stanton G. B. Bruce C. J. Goldberg M. E. (1995). Topography of projections to posterior cortical areas from the macaque frontal eye fields. *J. Comp. Neurol.* 353 291–305. 10.1002/cne.903530210 7745137

[B127] Stanton G. B. Deng S.-Y. Goldberg E. M. McMullen N. T. (1989). Cytoarchitectural characteristic of the frontal eye fields in macaque monkeys. *J. Comparative Neurol.* 282 415–427. 10.1002/cne.902820308 2715390

[B128] Stosiek C. Garaschuk O. Holthoff K. Konnerth A. (2003). In vivo two-photon calcium imaging of neuronal networks. *Proc. Natl. Acad. Sci. U.S.A.* 100 7319–7324. 12777621 10.1073/pnas.1232232100 PMC165873

[B129] Sul J. H. Jo S. Lee D. Jung M. W. (2011). Role of rodent secondary motor cortex in value-based action selection. *Nat. Neurosci.* 14 1202–1208. 10.1038/nn.2881 21841777 PMC3164897

[B130] Suriya-Arunroj L. Gail A. (2019). Complementary encoding of priors in monkey frontoparietal network supports a dual process of decision-making. *eLife* 8:e47581. 10.7554/eLife.47581 PMC6794075 31612855

[B131] Taghizadeh B. Foley N. C. Karimimehr S. Cohanpour M. Semework M. Sheth S. A. (2020). Reward uncertainty asymmetrically affects information transmission within the monkey fronto-parietal network. *Commun. Biol.* 3 1–11. 33087809 10.1038/s42003-020-01320-6 PMC7578031

[B132] Tanné-Gariépy J. Rouiller E. M. Boussaoud D. (2002). Parietal inputs to dorsal versus ventral premotor areas in the macaque monkey: evidence for largely segregated visuomotor pathways. *Exp. Brain Res.* 145 91–103. 10.1007/s00221-002-1078-9 12070749

[B133] Thal D. R. Rub U. Orantes M. Braak H. (2002). Phases of A beta-deposition in the human brain and its relevance for the development of AD. *Neurology* 58 1791–1800. 10.1212/wnl.58.12.1791 12084879

[B134] Uddin L. Q. Yeo B. T. T. Spreng R. N. (2019). Towards a universal taxonomy of macro-scale functional human brain networks. *Brain Topogr.* 32 926–942. 10.1007/s10548-019-00744-6 31707621 PMC7325607

[B135] Violante I. R. Li L. M. Carmichael D. W. Lorenz R. Leech R. Hampshire A. (2017). Externally induced frontoparietal synchronization modulates network dynamics and enhances working memory performance. *eLife* 6:e22001. 10.7554/eLife.22001 PMC5349849 28288700

[B136] Wardak C. Ibos G. Duhamel J.-R. Olivier E. (2006). Contribution of the monkey frontal eye field to covert visual attention. *J. Neurosci.* 26 4228–4235. 10.1523/jneurosci.3336-05.2006 16624943 PMC6674003

[B137] Weinrich M. Wise S. P. (1982). The premotor cortex of the monkey. *J. Neurosci.* 2 1329–1345. 7119878 10.1523/JNEUROSCI.02-09-01329.1982 PMC6564318

[B138] Westendorff S. Klaes C. Gail A. (2010). The cortical timeline for deciding on reach motor goals. *J. Neurosci.* 30 5426–5436. 10.1523/jneurosci.4628-09.2010 20392964 PMC6632761

[B139] Wickersham I. R. Lyon D. C. Barnard R. J. O. Mori T. Finke S. Conzelmann K.-K. (2007). Monosynaptic restriction of transsynaptic tracing from single, genetically targeted neurons. *Neuron* 53 639–647. 10.1016/j.neuron.2007.01.033 17329205 PMC2629495

[B140] Wilke M. Kagan I. Andersen R. A. (2012). Functional imaging reveals rapid reorganization of cortical activity after parietal inactivation in monkeys. *Proc. Natl. Acad. Sci. U.S.A.* 109 8274–8279. 10.1073/pnas.1204789109 22562793 PMC3361455

[B141] Wise S. P. Boussaoud D. Johnson P. B. Caminiti R. (1997). Premotor and parietal cortex: corticocortical connectivity and combinatorial computations. *Annu. Rev. Neurosci.* 20 25–42. 10.1146/annurev.neuro.20.1.25 9056706

[B142] Wise S. P. Murray E. A. (2000). Arbitrary associations between antecedents and actions. *Trends Neurosci.* 23 271–276. 10.1016/s0166-2236(00)01570-8 10838597

[B143] Yamashita T. Pala A. Pedrido L. Kremer Y. Welker E. Petersen C. C. (2013). Membrane potential dynamics of neocortical projection neurons driving target-specific signals. *Neuron* 80 1477–1490. 10.1016/j.neuron.2013.10.059 24360548

[B144] Zhang S. Xu M. Chang W.-C. Ma C. Hoang Do J. P. Jeong D. (2016). Organization of long-range inputs and outputs of frontal cortex for top-down control. *Nat. Neurosci.* 19 1733–1742. 10.1038/nn.4417 27749828 PMC5127741

[B145] Zhang S. Xu M. Kamigaki T. Do J. P. H. Chang W.-C. Jenvay S. (2014). Long-range and local circuits for top-down modulation of visual cortical processing. *Science* 345 660–665. 10.1126/science.1254126 25104383 PMC5776147

[B146] Zhao Q. Lu H. Metmer H. Li W. X. Y. Lu J. (2018). Evaluating functional connectivity of executive control network and frontoparietal network in Alzheimer’s disease. *Brain Res.* 1678 262–272. 10.1016/j.brainres.2017.10.025 29079506

[B147] Znamenskiy P. Zador A. M. (2013). Corticostriatal neurons in auditory cortex drive decisions during auditory discrimination. *Nature* 497 482–485. 10.1038/nature12077 23636333 PMC3670751

